# Identifying Patient Perceived Barriers to Trichiasis Surgery in Kongwa District, Tanzania

**DOI:** 10.1371/journal.pntd.0005211

**Published:** 2017-01-04

**Authors:** Ryan J. Bickley, Harran Mkocha, Beatriz Munoz, Sheila West

**Affiliations:** 1 Dana Center for Preventive Ophthalmology, Johns Hopkins University School of Medicine, Baltimore, Maryland, United States of America; 2 Kongwa Trachoma Project, Kongwa, Tanzania; University of California San Diego School of Medicine, UNITED STATES

## Abstract

**Background:**

Trachomatous trichiasis (TT), inturned eyelashes from repeated infection with *Chlamydia trachomatis*, is the leading infectious cause of blindness in the world. Though surgery will correct entropion caused by trachoma, uptake of TT surgery remains low. In this case-control study, we identify barriers that prevent TT patients from receiving sight-saving surgery.

**Methodology/Principal Findings:**

Participants were selected from a database of TT cases who did (acceptors) and did not (non-acceptors) have surgery as of August 2015. We developed an in-home interview questionnaire, using open and closed-ended questions on perceived barriers to accessing surgical services. We compared responses between the acceptors and non-acceptors, examining differences in reasons for and against surgery, sources of TT information, and suggestions for improving surgical delivery. 167 participants (mean age 61 years, 79.7% females) were interviewed. Compared to acceptors, non-acceptors were more likely to report they had no one to accompany them to surgery (75.3% vs. 42.6%, p<0.0001), they could manage TT on their own (69.9% vs. 31.5%, p<0.0001), and the surgery camp was too far (53.4% vs. 28.7%, p = 0.001). Over 90% of both acceptors and non-acceptors agreed on the benefits of having surgery. Fear of surgery was the biggest barrier stated by both groups. Despite this fear, acceptors were more likely than non-acceptors to also report fear of losing further vision without surgery.

**Conclusions/Significance:**

Barriers included access issues, familial and/or work responsibilities, the perception that self-management was sufficient, and lack of education about surgery. Fear of surgery was the biggest barrier facing both acceptors and non-acceptors. Increasing uptake will require addressing how surgery is presented to community residents, including outlining treatment logistics, surgical outcomes, and stressing the risk of vision loss.

## Author Summary

The burden of trachomatis trichiasis (TT), a complication of trachoma, is largest in the poorest societies of our world. One part of the World Health Organization (WHO) strategy to eliminate blinding trachoma is surgery for patients with TT, and trachoma programs in endemic countries have worked to increase the availability of surgical services. However despite these efforts, uptake of surgery remains low. In this study, we sought to identify the barriers that prevent TT patients from receiving sight-saving surgery. We interviewed residents with TT who did and did not have surgery. We found that fear was the biggest barrier among all participants, not just those who did not have surgery. But despite this fear, those who received surgery reported more often a fear of losing further vision had they not had surgery, highlighting that an understanding of the visual loss with TT is a possible motivating factor. Barriers to surgery included concern for going unaccompanied, and the perception that self-management was sufficient. Eliminating trachoma requires refining how surgery and education about the disease are presented to residents of endemic communities, including outlining treatment logistics, surgical outcomes, and the natural course of untreated TT.

## Introduction

Trachomatous trichiasis (TT), a complication of trachoma, is the leading infectious cause of preventable blindness in the world today [1]. Repeated infection of children with *Chlamydia trachomatis* can lead to scarring of the conjunctiva, causing entropion in adults as the eyelid turns inward, and trichiasis as the eyelashes deviate inward and touch the globe. Damage to the cornea from trichiasis, as well as abnormal conjunctiva, can result in vision loss. Trachoma remains a problem in the poorest societies of the world. As of 2014, an estimated 21 million people were afflicted with active trachoma, 7.3 million of whom have trichiasis and 2.2 million of whom are either completely blind or severely visually impaired. The majority of this blinding trachoma occurs in African countries [2].

The World Health Organization (WHO) recommends surgery to correct entropion caused by trachoma, and this has been shown to improve symptoms and even restore vision [3, 4]. Despite the benefits, uptake of TT surgery remains low in published reports [5–8]. Some work to elucidate barriers has been done [7, 9–14] but understanding differences between persons in the same communities who did and did not have surgery would clarify barriers and provide data to improve access to surgical programs.

Following a study on screening in Kongwa District, Tanzania for TT using Community Drug Distributors, a TT surgical camp was convened to offer free surgery and transport to the camp for all persons identified with TT [15]. Two years after the camp, we determined those who did and did not have surgery with the goal of interviewing them to identify barriers to receiving sight-saving surgery.

## Methods

### Ethics Statement

This research was approved by the Institutional Review Board of Johns Hopkins School of Medicine, and by the National Institute for Medical Research in Tanzania. The project was conducted according to the tenets of the Declaration of Helsinki. All subjects provided written, informed consent prior to participation.

### Population

We conducted a case-control study where cases were TT patients who did not have surgery, and controls were TT cases who did have surgery. TT cases were chosen from all 29 communities in Kongwa District, Tanzania who were in an existing database obtained during screening in 2013 [15]. Each case was confirmed by an eye nurse, and again by the first author, Mr. Bickley. There is no Master Grader training for trichiasis because the signs are so rare. For this study, Mr. Bickley’s training was provided using images and field practice sessions in Kongwa, following WHO criteria that trichiasis is at least one lash touching the globe or evidence of epilation. The training was conducted by the second author, Mr. Mkocha, who has 20 plus years’ experience grading trachoma and trichiasis in the field. The list of TT cases included those who had surgery during the camp, and those who did not have surgery. In addition to the surgery camp in 2013, static surgery services are available through the Kongwa District Hospital. Village health workers in these communities referred TT cases not on the list to participate in our study on the day of the interviews. The interviews were conducted from July 26 to August 19, 2015.

### Survey

Based on previous research [7, 9–14], we developed a questionnaire on perceived barriers to accessing health care and surgical services. The questionnaire was translated from English to Swahili and then back-translated from Swahili to English for comprehension, then pilot-tested in Kongwa in non-study villages and refined before administration in study villages.

**Fig 1:**
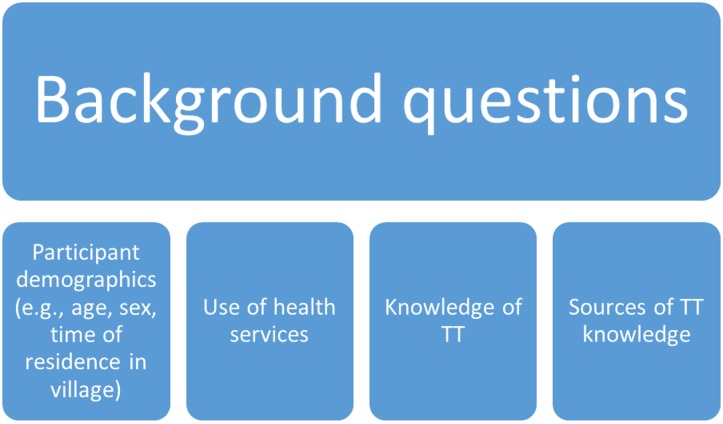
Questionnaire For trichiasis cases: background items.

The beginning of the questionnaire included the following domains: participant demographics, past use of health services, knowledge of TT, and source(s) of that knowledge (Fig 1: Background questions asked to participants).

**Fig 2:**
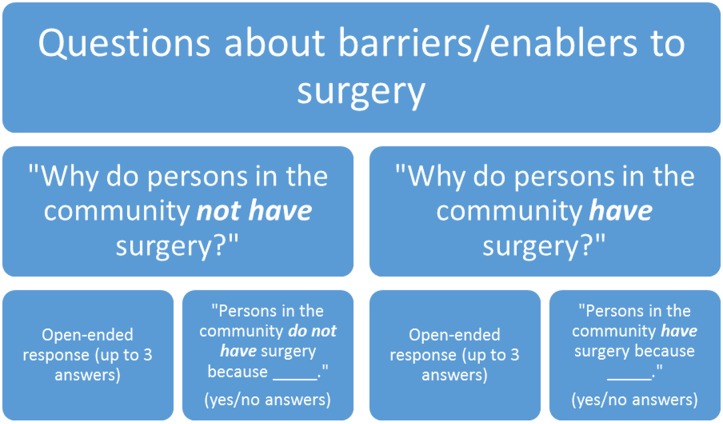
Questionnaire for trichiasis cases: items about factors that are perceived barriers or enablers to trichiasis surgery for community residents.

The next set of questions was designed to elicit barriers and enablers to surgery, and was performed in four parts: The first two parts asked for the perceptions of why “persons in the community” did not have surgery. The first set of questions was open-ended and the participant supplied answers, urged to provide up to three responses. The second set of questions required a yes or no response to a list of specific reasons that might prevent persons from having surgery. Third was a set of questions that asked open-ended questions to reasons why persons would have surgery, and this was followed by the fourth part, which was a set of questions eliciting yes or no responses to reasons why persons would have surgery (Fig 2: Questions about barriers/enablers to surgery asked to participants). For example, the question asking about barriers read as “Here are some of the reasons that we have heard are reasons that people with ‘*kope*’ [the local word for TT] do not go for surgery. Please tell us if you AGREE with these reasons, or if you do not think they are reasons why people with ‘*kope*’ do not have surgery”. In this way, the respondent does not have to state that this was his/her reason, but just that it was a reason people may not have surgery. We refer to these as “general” responses, rather than “personal” responses.

**Fig 3:**
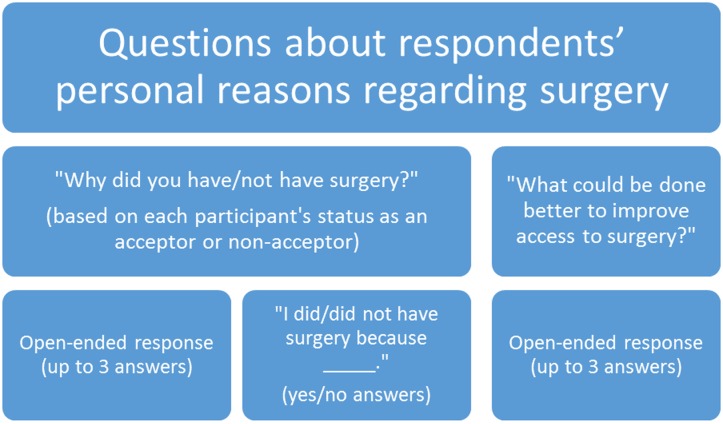
Questionnaire for trichiasis cases: items about factors that are perceived barriers or enablers to trichiasis surgery for the respondent in particular.

The last part of the interview asked about the respondents’ personal reasons for choosing to have or not have surgery, first using open-ended questions and then asking questions requiring a yes or no response. We refer to these as “personal” responses. The interview concluded with a question on perceptions of what could be done to improve access to and quality of surgical services in the district (Fig 3: Questions about respondents’ personal reasons regarding surgery).

In-home interviews were conducted by trained interviewers who spoke both Swahili and the local languages of Kigogo and Kikaguro. The interviewers were not aware of which participants had or had not had surgery until the end of the interview, at which point an envelope was opened and the status revealed. At that point the final piece of the interview was conducted.

Follow-up interviews were attempted at least 3 times for those TT cases that were temporarily away.

### Analysis

Data was entered in a customized Microsoft Access database and analyzed using SAS (Raleigh, NC). Using Fisher’s exact test, participant responses were compared between acceptors and non-acceptors of TT surgery. Specifically, differences were examined between demographics, use of health services, sources of learning about TT, reasons for and against having TT surgery (both specific to the participant and “general” to persons in the community), and suggested improvements for TT delivery to residents. Answers to free response questions were translated into English and recoded into categories before comparison.

A multivariate model with non-acceptance of surgery as an outcome adjusted for use of health services at the local post was constructed.

## Results

**Fig 4:**
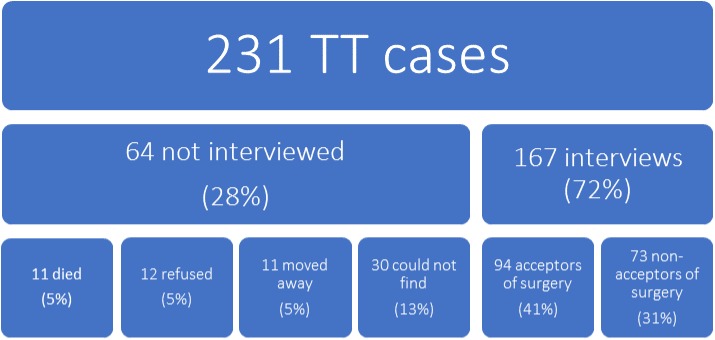
Participation and disposition of 231 trichiasis cases eligible to be in the study.

A total of 231 cases of TT were identified for this study. 179 TT cases were identified through the list created in 2012 [15] for surgery, and 52 were added at the time of interviewing in the village. Of the 179 cases from the 2012 list, 64 (28%) refused, died, or were not locatable. Nineteen of the 52 newly added cases were newcomers to their respective villages since 2012. The remaining 33 of those participants not on the 2012 list were in the original village censuses, but were not identified as TT cases at the time of the screening. Of 167 interviews, 93 were considered acceptors because they had surgery during the camp and 74 were considered non-acceptors because they did not have surgery (Fig 4: Final categorization of 231 TT cases identified for the study).

Most of the participants were female, and with a mean age above 60 years. There were no differences in age, gender distribution, time of residence in village, use of health services at Kongwa District Hospital, ability to identify their respective Community Health Worker, knowing anyone with TT, or knowing the health effects of having TT between acceptors and non-acceptors of surgery (Table 1). Non-acceptors were more likely to report use of health services at the local health post but less likely to report knowledge of the village surgical camp. Acceptors were more likely to have heard about TT from health personnel who provide trachoma MDA (Table 1).

**Table 1:** Characteristics of acceptors and non-acceptors of surgery.

Characteristic	Acceptors (N = 94)	Non-acceptors (N = 73)	p-value*
**Mean age in years (s.d.)**	60.7 (17.0)	62.0 (17.6)	0.61
**% Female**	82.0	76.7	0.33
**Mean years of residence in village (s.d.)**	44.3 (19.3)	45.6 (22.2)	0.69
**% use of health services at Kongwa District Hospital**	84.0	89.0	0.38
**% use of health services at local post**	36.6	60.3	0.003
**% correctly identify Community Health Worker**	27.8	23.5	0.59
**% know anyone with TT**	88.3	93.2	0.43
**% know that TT progresses to blindness**	95.7	87.7	0.08
**% knew about the surgical camp near village**	91.5	57.5	<0.0001

*Fisher’s exact test or T-test where appropriate

Table 2 evaluates the proportion, by surgical status, who responded affirmatively to closed-ended questions regarding barriers to having TT surgery as perceived by persons in the community. Significantly more non-acceptors reported that in “general” people state not being able to afford surgery (60.3%, p<0.001), not knowing where to receive surgery (39.7%, p = 0.0001), stating surgical camps were too far away (53.4%, p = 0.001), not having someone to accompany and help them (75.3%, p<0.0001), and claiming they could manage TT on their own with tweezers (69.9%, p<0.0001). Few respondents regardless of group indicated that reasons might include the possibility that surgery would not help, was done poorly, or resulted in poor appearance afterward.

**Table 2:** The “general” perceived barriers, compared between acceptors and non-acceptors of surgery when asked in closed-ended questions.

“General” perceived barrier to surgery	Acceptors (N = 94) % who said yes to this reason (n)	Non-acceptors (N = 73) % who said yes to this reason (n)	p-value*
**Do not need surgery**	38.3 (36)	31.5 (23)	0.42
**Do not know where to get surgery**	12.8 (12)	39.7 (29)	**0.0001**
**Cannot afford surgery**	19.6 (18)	60.3 (44)	**<0.001**
**Fear of surgery**	87.1 (81)	79.2 (57)	0.21
**Surgery location is too far away**	28.7 (27)	53.4 (39)	**0.001**
**Have no one to care for children or do work**	39.8 (37)	50.7 (37)	0.21
**Can manage TT by oneself**	31.5 (29)	69.9 (51)	**<0.0001**
**Have no one to go with patient and help**	42.6 (40)	75.3 (55)	**<0.0001**
**Surgery will not help TT**	2.1 (2)	6.9 (5)	0.24
**Surgeons do not do a good job**	9.6 (9)	6.9 (5)	0.59
**People who have surgery do not look good afterwards**	7.5 (7)	2.8 (2)	0.3

*Fisher’s exact test

Non-acceptors of surgery were asked about perceived barriers that others might report, and then asked directly about their own personal barriers. The answers they provided for reasons others might have given and their own reasons were compared (Table 3). Non-acceptors were more likely to report that others in “general” were afraid of getting surgery on their eyelids, 79.2%, versus reporting they personally had fears about surgery, 41.1% (p<0.0001). Similarly, non-acceptors were more likely to say that others did not have anyone to care for their children or complete their work while away at surgery (50.7% vs. 27.4%, p = 0.002), could manage TT on their own with tweezers (69.9% vs. 30.1%, p<0.0001), and did not have anyone to accompany them (the patient) to the surgery site (75.3% vs. 8.2%, p<0.0001), all compared to their personal reasons for not receiving surgery (Table 3). The most common reason that non-acceptors reported that others would give for not having surgery was fear. For themselves, however, the most common reasons reported was not being able to afford surgery, despite the fact that surgery as well as the transport to and from the camp were free. The second most common personal reason provided was not knowing where to get surgery, which may explain cost uncertainty.

**Table 3:** Among non-acceptors of surgery, the comparison of perceived “general” versus personal barriers to surgery.

Perceived barrier to surgery	“General” reasons (N = 73) % who said yes to this reason (n)	Personal reasons (N = 73) % who said yes to this reason (n)	p-value*
**Do not need surgery**	31.5 (23)	24.7 (18)	0.27
**Do not know where to get surgery**	39.7 (29)	43.8 (32)	0.53
**Cannot afford surgery**	60.3 (44)	48.0 (35)	0.11
**Fear of surgery**	79.2 (57)	41.1 (30)	**< 0.0001**
**Surgery location is too far away**	53.4 (39)	38.4 (28)	0.10
**Have no one to care for children or do work**	50.7 (37)	27.4 (20)	**0.002**
**Can manage TT by oneself**	69.9 (51)	30.1 (22)	**< 0.0001**
**Have no one to go with patient and help**	75.3 (55)	8.2 (6)	**< 0.0001**
**Surgery will not help TT**	6.9 (5)	5.6 (4)	0.48
**Surgeons do not do a good job**	6.9 (5)	1.4 (1)	0.10
**People who have surgery do not look good afterwards**	2.8 (2)	1.4 (1)	0.56

*McNemar’s test for correlated proportions

Women were significantly more likely than men to report a “general” barrier was no one to accompany them to surgery. When asked about personal reasons, women were significantly more likely to report that they could manage TT with tweezers compared to males. Otherwise, the answers were comparable across genders.

The open-ended question where participants could provide reasons why persons did not have surgery was not answered by 15%, and the most common answers had fear as a major component for both groups (Table 4).

**Table 4:** The perceived “general” barriers, compared between acceptors and non-acceptors of surgery when asked in an open-ended fashion.

“General” perceived barrier to surgery	Acceptors (N = 94) % who gave this reason (n)	Non-acceptors (N = 73) % who gave this reason (n)
**No reason**	14.9 (14)	15.1 (11)
**Lack of education**	16.0 (15)	21.9 (16)
**Do not need surgery**	0.0 (0)	1.4 (1)
**Do not know where to go to get surgery**	0.0 (0)	1.4 (1)
**Cannot afford surgery**	1.1 (1)	1.4 (1)
**Fear of surgery**	21.3 (20)	21.9 (16)
**Fear (nonspecific)**	31.9 (30)	21.9 (16)
**Fear of having eyes ruined**	8.5 (8)	6.9 (5)
**Fear of pain and/or avoidance of pain**	3.3 (3)	2.7 (2)
**Fear of being unable to work after surgery**	0.0 (0)	2.7 (2)
**Avoid blindness**	1.1 (1)	0.0 (0)
**Inopportune time because of work/family issues**	1.1 (1)	1.4 (1)
**Have no one to go with patient and help**	0.0 (0)	1.4 (1)
**Surgery will not help TT**	1.1 (1)	0.0 (0)

When participants were asked about the perceived benefits of having surgery for persons in “general” in an open-ended question, responses were similar between acceptors and non-acceptors, except that non-acceptors were less likely to spontaneously report a benefit to seeing better after surgery/going blind without surgery (Table 5). This finding contrasts to the benefits elicited when asked in a closed-ended fashion, where there were no significant differences between responses of acceptors and non-acceptors (Table 6). All the reasons, apart from simply being told to have surgery, appeared to be important reasons for surgery as reported by both groups.

**Table 5:** The perceived “general” benefits of having surgery, compared between acceptors and non-acceptors of surgery when asked using an open-ended question.

“General” perceived benefit of surgery	Acceptors (N = 94) % who gave this reason (n)	Non-acceptors (N = 73) % who gave this reason (n)
**No reason**	3.2 (3)	2.7 (2)
**Get rid of pain/discomfort/tearing**	31.9 (30)	34.3 (25)
**See better after surgery/go blind without surgery**	47.9 (45)*	30.1 (22)*
**Cannot manage TT by oneself**	1.1 (1)	0.0 (0)
**Cure/help eyes (nonspecific, not mentioning vision)**	10.6 (10)	21.9 (16)
**To fix the problem (nonspecific, not mentioning eyes)**	5.3 (5)	11.0 (8)

*p<0.02

**Table 6:** The perceived “general” benefits of having surgery, compared between acceptors and non-acceptors of surgery when asked using closed-ended questions.

“General” perceived benefit of surgery	Acceptors (N = 94) % who said yes to this reason (n)	Non-acceptors (N = 73) % who said yes to this reason (n)	p-value*
**Get rid of the pain**	97.9 (92)	98.6 (72)	1.0
**See better after surgery**	97.9 (92)	97.3 (71)	1.0
**Avoid blindness**	97.8 (90)	97.3 (71)	1.0
**Person was told to have surgery**	22.3 (21)	19.2 (14)	0.70
**Cannot manage TT by oneself**	92.6 (87)	94.5 (69)	0.76

*Fisher’s exact test

Both groups of participants were asked for their opinion on what might be done to improve access to surgical services supplied by the district (Table 7). Each participant could submit up to three responses. For non-acceptors, the most frequent response was that surgical efforts should continue, and that efforts to continue to aid village residents were needed. For acceptors, the most common suggestion was to provide more education and advice about the need for surgery. Of note, acceptors were more likely to suggest that successful patients should be examples for others, and non-acceptors were more likely to propose providing better information on surgical availability.

**Table 7:** Suggested improvements to surgical care delivery, compared between acceptors and non-acceptors.

Suggestion for improvement	Acceptors (N = 94) % who gave this reason (n)	Non-acceptors (N = 73) % who gave this reason (n)
**No response**	17.1 (19)	15.2 (12)
**Continue doing surgical work**	21.6 (24)	43.0 (34)
**Provide education/advice about surgery**	26.1 (29)	16.5 (13)
**Provide follow-up after surgery**	13.5 (15)	7.6 (6)
**Have successful patients be examples**	9.0 (10)	1.3 (1)
**Visit TT patients at home**	4.5 (5)	1.3 (1)
**Do better surgical work**	3.6 (4)	3.8 (3)
**Help with other eye problems, not just TT**	2.7 (3)	1.3 (1)
**Better inform people of surgery availability**	0.0 (0)	5.1 (4)
**Provide incentives for having TT surgery**	0.9 (1)	1.3 (1)
**Try to find more people with TT**	0.9 (1)	1.3 (1)
**Come help others, but not me**	0.0 (0)	2.5 (2)
Total number of answers*	*100*.*0 (111)*	*100*.*0 (79)*

*participants could submit up to 3 responses

A multivariate model with non-acceptance of surgery as an outcome adjusted for use of health services at the local post found the same predictors as are reported here in the univariate models.

## Discussion

Though the WHO recommends surgery for TT to prevent progression to blindness, uptake of surgery remains a problem. Previous studies in Kongwa among non-acceptors in the mid-1990s identified barriers to uptake as transportation to surgical sites, cost of surgery, arranging for the care of family and/or farm work while the patient is indisposed, and having someone to accompany the patient to the surgery site [9, 11], responses that we found continue to be important. At that time, the only surgical services were provided by local non-governmental organizations (NGOs) in the local hospital and there were no community-based surgical camps. Despite trying to bring free services closer and providing transport to and from the camps, the perception of access barriers to having surgery remain.

Our results are also similar to those found in a 2012 study in Ethiopia, which identified the greatest barriers as financial, both direct and indirect [10]. Though surgery camps in our study region of Kongwa District, Tanzania were provided free of charge, patients still needed to take time off their farm work and childcare during the day of surgery and recovery, leading to an indirect opportunity cost to having TT surgery. Opportunity cost is not an insubstantial component in evaluating efficiency of health care delivery. Although providers see the ten minutes of a procedure as an investment, many patients are faced with a loss of several days that they may not be able to afford, especially if the patient is female with no child care options.

We used a variety of approaches to elicit responses on reasons for accepting or not accepting surgery, including asking about reasons people in “general” might have and reasons participants in personally had for choosing or not choosing to have surgery. Despite using local interviewers when administering our questionnaire, it is possible that participants saw us as connected to health providers simply by asking questions about health care delivery. Because health services in our study areas are so limited, participants may have censored their responses so as not to seem critical or ungrateful of the efforts of health providers. We felt that by allowing participants first to answer what people in “general” might say are barriers, we were in essence eliciting responses they were hesitant to attribute to themselves. This was a valuable approach, as exemplified by our finding that fear was a major barrier to receiving surgery for both acceptors and non-acceptors. Among non-acceptors of surgery, fear was more often reported as a “general” reason against surgery, and not a personal reason for that individual. The fact that fear was a recurring reason in free response answers as well as an affirmative response to a forced choice question validates it as an important reason. It is notable that fear was not among the reasons cited for non-acceptance in earlier studies in Kongwa, although one response at that time was “I would not have surgery under any condition,” of which fear may be a strong component.

If both groups stated that fear motivated them at least to some extent on their choice whether or not to have surgery, what were the factors that enabled the acceptors of surgery to overcome their own fears and receive surgery? When first asked in an open-ended fashion why others in “general” do have surgery, significantly more acceptors spontaneously responded that people feared losing their vision if they did not receive the surgery. When provided this response as a close ended question, 97% in both groups responded that this was a reason for surgery, although the non-acceptors were less likely to identify it on their own. We suggest that the issue of seeing better after/going blind without surgery was more on the forefront of acceptors’ minds, and was greater than the fear of the surgery itself. This difference in perceived benefits could also indicate a motivating factor for acceptors, and might suggest that more efforts need to be placed on education so that those resistant to having surgery fully understand the consequences of their disease.

One limitation of our study is that severity of TT was not measured. That data would be useful to understanding the differential response to the fear of losing vision as a consequence of not having surgery. Our finding that non-acceptors were more likely to report they could manage their TT themselves with epilation may also indicated less severe TT in this group compared to the acceptors.

We note that 26% of acceptors of surgery suggested that better education and advice about the surgery would help improve services. A 2015 study in Egypt that sought methods of increasing surgical uptake demonstrated that community-based eye education programs greatly increased the uptake of TT surgery among residents [16]. In our study, a high proportion of both acceptors and non-acceptors had knowledge of what eventually happens to eyes with TT, but the fear of losing vision was a stronger motivator among the acceptors. How to translate the knowledge into action appears to be crucial. Certainly better knowledge about what the surgery actually entails would allow patients to not only understand the course of their disease, but have a better understanding of the treatment. Acceptors suggested the use of successful surgery patients as ambassadors in villages in order to explain to patients the process of surgery, including how they (the ambassadors) were able to overcome their personal fears of having surgery. Though this finding may be seen as self-serving, successful surgery patients could be strong voices to help convince non-acceptors to receive surgery; hesitant patients may be more willing to trust fellow villagers as opposed to outsiders to their community. Cataract surgery motivators from within the community have been used in India in previous studies [17, 18]. Another study in India that examined barriers to surgery for TT also recommended the use of successful patients sharing their experiences with other afflicted community members, in addition to making educational materials more available [12]. As non-acceptors report presenting frequently to local health posts, these sites could be suitable as intervention points for public health education regarding the disease of TT and its surgical treatment. Because of a lack of concern among participants for the quality or efficacy of the surgery, efforts to increase uptake must center on what constitutes fear and how it can be overcome, as opposed to convincing residents of surgeons’ quality or proficiencies. Having local villagers who have undergone successful surgery present and share their stories may go a long way in convincing residents who were previously afraid of surgery.

Misinformation around the surgery adds to perceived barriers. Non-acceptors stated the cost of surgery and the location of surgery were both barriers, despite the surgery being offered for free and despite transportation being provided to surgery sites. Some of these responses were due to the higher proportion of newcomers to the village among non-acceptors, and so they would naturally be less likely to have been present or had the information when the surgical camps occurred. Nevertheless, a high proportion of those who had surgery said they heard this was a barrier in their communities, indicating a problem of misinformation. There is a new initiative in Kongwa to treat TT surgery with camps, so as more camps occur, this problem should decline with proper advance communication to residents. Clearly, more efforts should be spent on spreading knowledge of upcoming dates of the surgical camps, including publicizing that they are free of charge and that transport will be provided. Not only will residents better understand where the camps are occurring and at no cost to them, but patients with TT will be able to prepare by finding others to help manage childcare and/or farm and work responsibilities along with someone to help accompany them for their surgery.

Outcomes studies are in agreement with the perception that patients who underwent TT surgery have their symptoms alleviated and avoid vision loss, and others have shown improvements in quality of life [19–22]. Additionally, women tend to be more affected by TT than men, since women traditionally care for and therefore gain more exposure to infected children [7, 23]. Overall, 79% of our study population was female, although there was no evidence of gender bias in who accepted or did not accept surgery. A study in Niger examined how TT affected the quality of life of women and found that the associated sharp pain in the eyes, embarrassment and stigma from friends, family, and their communities, as well as an inability to travel, work, and earn an income were all significant negative effects of having the disease [13]. Increasing the uptake of TT surgery will have the added benefit of reducing gender-based health disparities in endemic communities, in addition to reducing the burden of TT.

There are other limitations to our study. While most residents that were approached agreed to participate, 12 (6.7% of total residents encountered) refused to be interviewed. All who refused had not received TT surgery, introducing potential bias, since their reasons for non-acceptance of surgery may be different than the reasons of those who did agree to be interviewed. While we cannot estimate this, the fact that the refusal rate was only 6.7% suggests bias would be minimal.

We had specific hypotheses about the barriers in each of the domains, but recognize that some significant associations may be based on chance alone. Our findings are bolstered by the consistency between the open- and close-ended responses.

There may be differences by community as well, in strength of education about TT, cultural beliefs about surgery and TT, or other factors. We surveyed too few persons with TT in each village to adjust by village, but we tried to balance this shortage by including in our study acceptors and non-acceptors in each village.

There is a limitation in generalizing our findings beyond the experience in Kongwa District, Tanzania. However, some of our findings are very similar to other reports from Ethiopia, Nigeria, The Gambia, India, and other districts in Tanzania [7, 10, 12–14], suggesting the usefulness of applying our findings to other settings.

Our study had several advantages over previous studies. First, we used a variety of approaches to elicit responses, which when concordant strengthened the likelihood the responses were valid. Non-acceptors may be reluctant to admit true reasons for not having surgery, and by asking them to report for people in “general” lessoned the discomfort in responding. This indirect method of interviewing allowed for participants to answer as honestly as possible while trying to minimize any hesitancy at being candid. In addition, the fact that we interviewed participants at home and used experienced female interviewers who spoke the local languages allowed an environment in which participants could speak as freely as possible. Second, we elicited responses from both those who did and did not have surgery among those who were eligible for surgery in order to determine the differences that could be drivers of non-acceptance. Most previous studies have confined questions to those who did not accept surgery only or do not differentiate between those who accept and did not accept surgery [5, 7, 10–14]. Our comparison could then focus on barriers that are more unique to non-acceptors rather than those that are overall barriers.

Our study on barriers to surgery suggests that eliminating blinding trachoma will require addressing how surgery is presented to community residents, including outlining logistics of treatment, surgical outcomes, follow-up plans, as well as alternatives and the natural course of non-treated TT. Fear of surgery is a major barrier facing both TT patients who did and did not have surgery, not just non-acceptors. We noticed a reluctance among participants to admit their true feelings toward surgery, and future studies of barriers to care must be sensitive to this. One idea worth further exploration is having successful surgical patients serve as ambassadors in their communities to explain the benefits and process of surgery to others with TT.

## Supporting Information

S1 Checklist STROBE file. (PDF)
